# Common Bile Duct Obstruction by Free Floating Tumor

**DOI:** 10.1155/1993/25314

**Published:** 1993

**Authors:** Richard A. Prinz, Tien C. Ko, Sheldon B. Maltz, Carlos J. Reynes, Richard E. Marsan, Robert J. Freeark

**Affiliations:** Departments of Surgery and Radiology Loyola University Medical Center 2160 South First Avenue Maywood Illinois 60153 USA

## Abstract

Tumors usually spread by local invasion or by vascular or lymphatic metastases. We report six patients in whom tumor cells were shed into the common bile duct with resulting obstruction. The three men and three women had jaundice and upper abdominal discomfort. Jaundice was intermittent in four patients. Preoperative total serum bilirubin ranged from 2.5 to 16.1 mg/dl; alkaline phosphatase ranged from 221 to 605 IU/1. Ultrsasound showed a dilated gallbladder [GB] in five patients with dilated intrahepatic ducts in three and stones in only one. ERCP showed a single filling defect in two of three patients and multiple defects in one. PTC showed multiple defects in one patient. At operation a thick gelatinous tissue fragment or clot was seen in the common bile duct of each patient. Frozen section identified tumor tissue in all. The source was GB carcinoma [2], GB adenomyoma [1], hepatic metastases of colon cancer [2] and common bile duct cancer [1]. Treatment consisted of pancreaticoduodenectomy [2], including one for GB cancer, left hepatic lobectomy [1], choledochoduodenostomy [1], common duct exploration with T-tube insertion and cholecystectomy [1]. One patient with metastatic colon cancer and another with gallbladder cancer died within one year of operation. The other four are alive from 2 to 4 years later. Conclusion: Benign or malignant tumors within the hepatobiliary tree can shed tissue into the common bile duct which can cause biliary obstruction. Any tissue fragment found in the common bile duct should be evaluated by frozen section. Recognition of this mode of tumor spread is needed for appropriate therapy of the underlying benign or malignant tumor.


*HPB Surgery*, 1993, Vol. 6, pp. 319–323
Reprints available directly from the publisher
Photocopying permitted by license only

© 1993 Harwood Academic Publishers GmbH
Printed in the United States of America

### CASE REPORT

### COMMON BILE DUCT OBSTRUCTION BY FREE FLOATING TUMOR

RICHARD A. PRINZ, TIEN C. KO, SHELDON B. MALTZ, CARLOS J. REYNES, RICHARD E. MARSAN AND ROBERT J. FREEARK

*Departments of Surgery and Radiology, Loyola University Medical Center, Maywood, Illinois, USA*

*(Received 27 January 1992)*

Tumors usually spread by local invasion or by vascular or lymphatic metastases. We report six patients in whom tumor cells were shed into the common bile duct with resulting obstruction. The three men and three women had jaundice and upper abdominal discomfort. Jaundice was intermittent in four patients. Preoperative total serum bilirubin ranged from 2.5 to 16.1 mg/dl; alkaline phosphatase ranged from 221 to 605 IU/1. Ultrsasound showed a dilated gallbladder [GB] in five patients with dilated intrahepatic ducts in three and stones in only one. ERCP showed a single filling defect in two of three patients and multiple defects in one. PTC showed multiple defects in one patient. At operation a thick gelatinous tissue fragment or clot was seen in the common bile duct of each patient. Frozen section identified tumor tissue in all. The source was GB carcinoma [2], GB adenomyoma [1], hepatic metastases of colon cancer [2] and common bile duct cancer [1]. Treatment consisted of pancreaticoduodenectomy [2], including one for GB cancer, left hepatic lobectomy [1], choledochoduodenostomy [1], common duct exploration with T-tube insertion [1] and cholecystectomy [1]. One patient with metastatic colon cancer and another with gallbladder cancer died within one year of operation. The other four are alive from 2 to 4 years later. Conclusion: Benign or malignant tumors within the hepatobiliary tree can shed tissue into the common bile duct which can cause biliary obstruction. Any tissue fragment found in the common bile duct should be evaluated by frozen section. Recognition of this mode of tumor spread is needed for appropriate therapy of the underlying benign or malignant tumor.

KEY WORDS: Bile, duct, obstruction, jaundice

In the gastrointestinal tract, tumor cells or tissue fragments can be shed from the serosal or mucosal surfaces and implant transperitoneally or intraluminally. This latter type of spread can occur with tumors in the hepatobiliary tree. Sloughing of tumor tissue or cells into the bile ducts can cause obturator obstruction of the common bile duct[1-4]. Patients with this problem can present with obstructive jaundice and/or biliary colic. Since this is extremely unusual both as a mode of tumor spread and as a cause of obstructive jaundice, the potential for error in diagnosis and management is high. This report reviews the clinical features of six patients in whom tumor cells or tissue fragments were shed into the common bile duct with resulting intermittent or constant biliary tract obstruction.

Address correspondence to: Richard A. Prinz, M.D., Loyola University Medical Center, 2160 South First Avenue, Maywood, Illinois 60153, USA

### PATIENTS AND METHODS

Between 1979 and 1989, six patients with tumor tissue fragments in the common bile duct were treated on the surgical service at Loyola University Medical Center. The group consists of three men and three women ranging in age from 41 to 88 years. Their mean age was 64 years. Three patients complained of recurrent upper abdominal pain while three patients complained of bloating with meals. Each patient had jaundice. The jaundice was intermittent in four patients and progressive in two. In two patients with intermittent jaundice, the bilirubin returned to normal levels.

Preoperative total serum bilirubin ranged from 2.5 to 16.1 mg/dl with a mean of 6.0 mg/dl (Normal serum bilirubin is 0.2 to 1.5 mg/dl). Serum alkaline phosphatase ranged from 221 to 605 IU/1 with a mean of 417 IU/1 (Normal serum alkaline phosphatase is 30 to 110 IU/1). Ultrasound of the gallbladder showed a dilated gallbladder without stones in five patients. One of these had a thickened wall indicative of chronic inflammation. Gallstones were noted in one patient. Endoscopic retrograde cholangiopancreatography (ERCP) was performed in three patients. It showed a single filling defect in one patient (Figure 1) and multiple filling defects in two. Percutaneous transhepatic cholangiography (PTC) was done in one patient and showed multiple filling defects. The multiple filling defects were noted throughout the extrahepatic biliary tree.

### RESULTS

All six patients underwent operation. In five the indication for surgery was jaundice and abdominal discomfort. One patient who was febrile and had an elevated white blood cell count, was operated on for cholangitis. At operation, a thick gelatinous tissue fragment or clot was found in the common bile duct of each patient. The source of this sloughed tissue was a benign adenomyoma of the gallbladder in one patient, adenocarcinoma of the gallbladder in two patients, hepatic metastases of adenocarcinoma of the colon in two patients and adenocarcinoma of the distal common bile duct in one patient. The tumor ranged from 4 mm to 15 mm in size. In two patients, one with gallbladder carcinoma and the other with metastatic colon cancer, the tumor fragment was large enough to replace almost the entire common duct. In the remaining four, the tumor embolus was less than a centimeter in size and appeared almost like a small bit of old blood clot.

The benign adenomyoma of the gallbladder was treated by cholecystectomy. Intraoperative cholangiography showed no evidence of a filling defect that had been seen on a preoperative ERCP. This patient remains well 2.5 years after cholecystectomy. A common bile duct exploration was not performed because of the normal intraoperative cholangiogram. The mucosal surface of the residual adenomyoma in the gallbladder was ulcerated. Apparently, a tissue fragment had sloughed and passed spontaneously through the cystic duct and common duct into the duodenum. Two patients had pancreaticoduodenectomy or Whipple resection. The first was operated on for obstructive jaundice in 1979. Common bile duct exploration revealed a free floating gelatinous tissue clot in the distal common bile duct. Frozen sections showed a well differentiated adenocarcinoma. A Whipple resection was performed which revealed a small carcinoma originating in the distal common bile duct. This patient was alive and well 3.5 years after operation but has been subsequently lost to follow-up. The other patient had a cholecystectomy and common bile duct exploration for a filling defect seen within the common bile duct on ERCP. (Figure 1) A 0.5 cm tissue fragment was seen floating in the common duct and sent for frozen section. This revealed well differentiated adenocarcinoma. Choledochoscopy revealed inflammation of the distal common bile duct. Whipple resection was performed to rule out and treat further tumor. Permanent evaluation revealed a well differentiated adenocarcinoma of the gallbladder and no other tumor in the pancreaticoduodenectomy specimen. This patient is alive and well four years after operation. The other patient with a gallbladder carcinoma had a cholecystectomy and choledochoduodenostomy but died ten months after operation from local progression of disease. This was the only patient in this series with gallstones. Two patients had metastatic adenocarcinoma of the colon. The first patient presented four years earlier with a lesion in the sigmoid colon and underwent a low anterior resection of the sigmoid for a modified Dukes' or Astler-Coller stage C-2 tumor. This patient had a left hepatic lobectomy, cholecystectomy and common bile duct explorastion and remains well three years after operation. The other patient with metastatic adenocarcinoma of the colon also presented four years after having a right hemicolectomy for a well differentiated mucinous adenocarcinoma, modified Dukes' or Astler-Coller stage C-2. At operation, there was extensive peritoneal seeding. The common bile duct contained a thick gelatinous material which formed a cast of the bile duct lumen. The common bile duct was drained with a T-tube because of the advanced state of disease. This patient died two months later.

### DISCUSSION

Biliary obstruction due to shedding of tumor cells or tissue fragments into the bile ducts was reported in two patients by Rudstom in 1951[1]. The first patient had sloughing of a primary hepatocellular carcinoma while the second patient had sloughing of a malignant melanoma metastatic to the gallbladder. This initial report pointed out that both primary and metastatic tumors within the hepatobiliary tree could cause obturator obstruction of the common bile duct.

Obturator obstruction of the biliary tree has been most commonly reported with hepatocellular carcinoma[2-9]. Lee and co-workers reported that 71 of 188 patients with hepatocellular carcinoma presented with jaundice[10]. The cause was most often liver replacement by tumor and associated cirrhosis. However, 22 of the 71 had obstructive jaundice and ten of these had a filling defect within the common bile duct. The authors did not have pathologic confirmation of the filling defects in these ten patients but suggested that they were due either to hemorrhage into the biliary tree or direct extension with sloughing of tumor into the biliary tree.

The present series of six patients indicates that both benign and malignant and primary and metastatic tumors can be shed into the bile ducts and cause common bile duct obstruction. One patient with gallbladder carcinoma presented with cholangitis. At operation a large tissue clot was identified obstructing the distal common bile duct. Wind and Futterman have reported a patient presenting with cholangitis that had a tissue slough in the common bile duct from a hepatoma[11].

Most patients with metastatic tumors to the liver develop jaundice from hepatic

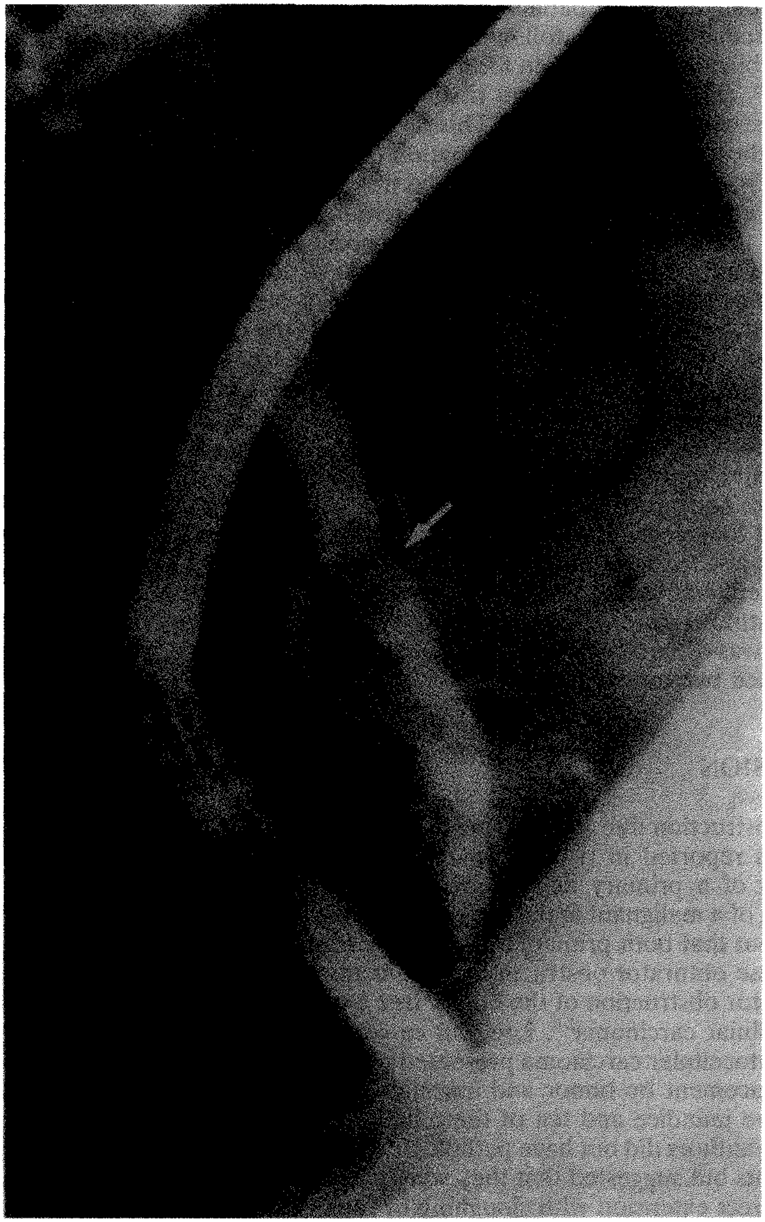

**Figure 1** ERCP shows a solitary filling defect due to a tumor embolus from a well differentiated adenocarcinoma of the gallbladder.

parenchymal destruction or extrinsic compression of the bile ducts. As noted above one of the first patients presenting with tumor tissue floating in the common bile duct had a malignant melanoma[1]. Verbanck and co-workers have reported a primary melanoma of the gallbladder that was shed into the common bile duct[12].

We report two patients with colon cancer and obturator obstruction of the biliary tree illustrating that a variety of tumors that metastasize to the liver can cause this problem.

Since obturator obstruction of the common bile duct is an unusual cause of obstructive jaundice and since most tumors cause jaundice by other means, the potential for error in diagnosis and treatment is real. This is shown by the patient with gallbladder carcinoma who underwent a Whipple resection to be certain there was no residual tumor in the distal-bile ducts. To avoid this type of error, it is important to remember that tumor cells or tissue fragments can be shed into the common bile duct from primary, benign or malignant tumors or metastatic tumors anywhere within the hepatobiliary tree. These tumor fragments can then cause common bile duct obstruction. Any tissue fragment in the common bile duct should be evaluated by frozen section. These tissue fragments are often very unimpressive and appear grossly like a blood clot or degenerated debris. Recognition of this mode of tumor spread is needed for appropriate therapy of the tumor and the associated jaundice. Long term survival is possible with appropriate therapy even in patients with metastatic disease.

### *Acknowledgement*

We would like to thank Linda Wahrer for her assistance in typing the manuscript, and Sharon Arizzi and Pamela Wielgos for their assistance in the preparation of the illustration.

### *References*

1. Rudstom, P. (1951) Hemobilia in malignant tumours of the liver. *Acta Chir.Scand.*, **101**, 243–246
2. Afroudakis, A., Bhuta, S.M., Ranganath, K.A. and Kaplowitz, N. (1978) Obstructive jaundice caused by hepatocellular carcinoma: Report of three cases. *Amer.J.of Dig.Dis.*, **23**, 609–617
3. Brand, S.N., Brandt, L.J., Sprayregan, S., Brenner, S. and Bernstein, L.H. (1976) Extrahepatic biliary tract obstruction secondary to a hepatoma-containing blood clot in the common bile duct. *Amer. J. of Dig.Dis.*, **21**(10), 905–909
4. Fisher, E.R. and Creed, D.L. (1956) Clot formation in the common duct. *Arch.of Surg.*, 261–265
5. Johns, W.A. and Zimmerman, A. (1961) Biliary obstruction due to hemobilia caused by liver cell carcinoma. *Ann.Surg.*, **153**(5), 706–710
6. Maffessanti, M.M., Bazzochi, M. and Melato, M. (1982) Sonographic diagnosis of intraductal hepatoma. *J.Clin.Ultrasound*, **10**, 397–399
7. Nonomura, A., Ohta, G., Kanai, M. and Kobayashi, K. (1983) Hepatocellular carcinoma presenting extrahepatic biliary obstruction. *Acta Pathol.Jpn.*, **33**(4), 789–806
8. Van Sonnenberg, E. and Ferrucci, J.T. (1979) Bile duct obstruction in hepatocellular carcinoma (hepatoma) — clinical and cholangiographic characteristics. Report of 6 cases and review of the literature. *Radiology*, **130**, 7–13
9. Tsuzuki, T., Ogata, Y., Iida, S., Kasajima, M. and Takahashi, S. (1979) Hepatoma with obstructive jaundice due to the migration of a tumor mass in the biliary tract: Report of a successful resection. *Surg.*, **85**(5), 593–598
10. Lee, N.W., Wong, K.P., Siu, K.F. and Wong, J. (1984) Cholangiography in hepatocellular carcinoma with obstructive jaundice. *Clin.Radiology*, 119–123
11. Wind, G. and Futterman, S. (1977) Obstructive jaundice secondary to hepatoma. *Amer.J.Gastroenterology*, **67**(1), 80–83
12. Verbanck, J.J., Rutgeerts, L.J., Van Aelst, F.J., Tytgat, J.H., Decoster, J.M., Noyez, D.N., Theunynck, P.J. and Geboes, K.J. (1986) Primary malignant melanoma of the gallbladder, metastatic to the common bile duct. *Gastroenterology*, **91**, 214–218

*(Accepted by S. Bengmark 28 January 1991)*

